# Ciprofloxacin-Eluting Nanofibers Inhibits Biofilm Formation by *Pseudomonas aeruginosa* and a Methicillin-Resistant *Staphylococcus aureus*

**DOI:** 10.1371/journal.pone.0123648

**Published:** 2015-04-08

**Authors:** Jayesh J. Ahire, Deon P. Neveling, Melanie Hattingh, Leon M. T. Dicks

**Affiliations:** Department of Microbiology, University of Stellenbosch, Matieland (Stellenbosch), South Africa; Ghent University, BELGIUM

## Abstract

*Pseudomonas aeruginosa* and *Staphylococcus aureus* are commonly associated with hospital-acquired infections and are known to form biofilms. Ciprofloxacin (CIP), which is normally used to treat these infections, is seldom effective in killing cells in a biofilm. This is mostly due to slow or weak penetration of CIP to the core of biofilms. The problem is accentuated by the release of CIP below MIC (minimal inhibitory concentration) levels following a rapid (burst) release. The aim of this study was to develop a drug carrier that would keep CIP above MIC levels for an extended period. Ciprofloxacin was suspended into poly(D,L-lactide) (PDLLA) and poly(ethylene oxide) (PEO), and electrospun into nanofibers (CIP-F). All of the CIP was released from the nanofibers within 2 h, which is typical of a burst release. However, 99% of *P*. *aeruginosa* PA01 cells and 91% of *S*. *aureus* Xen 30 cells (a methicillin-resistant strain) in biofilms were killed when exposed to CIP-F. CIP levels remained above MIC for 5 days, as shown by growth inhibition of the cells *in vitro*. The nanofibers were smooth in texture with no bead formation, as revealed by scanning electron and atomic force microscopy. A single vibration peak at 1632 cm^-1^, recorded with Fourier transform infrared spectroscopy, indicated that CIP remained in crystal form when incorporated into PDLLA: PEO. No abnormalities in the histology of MCF-12A breast epithelial cells were observed when exposed to CIP-F. This is the first report of the inhibition of biofilm formation by CIP released from PDLLA: PEO nanofibers.

## Introduction

*Pseudomonas aeruginosa* and *Staphylococcus aureus* are amongst the most dominant pathogens isolated from nosocomial infections [1, 2]. Continuous exposure to sub-lethal concentrations of antibiotics and the ability of cells to form biofilms may lead to the development of antibiotic-resistant cell populations [3]. Biofilm formation is often enhanced by the production of exopolysaccharides (EPS) [4]. Synergistic growth of *P*. *aeruginosa* and *S*. *aureus* alter virulence and delay wound healing [5]. The exclusion of *P*. *aeruginosa* and *S*. *aureus* from wounds is thus of clinical importance.

Recent developments in nanotechnology opened new possibilities in infection control. Specialised nanofiber dressings have more advantages compared to normal dressings such as gauzes and bandages [6]. Nanofiber wound dressings not only provide protection of the wound from mechanical trauma, bacterial infiltration, gaseous and fluid exchange, but also favours sustained release of antimicrobial compounds [6–8]. Nanofibers prepared from combinations of hydrophobic poly(D,L-lactide) (PDLLA) and hydrophilic poly(ethylene oxide) (PEO) are biocompatible and have been approved by the Food and Drug Administration (FDA) for incorporation into medical implants by [9, 10]. Reports on the controlled release of bacteriocins, chelators and drugs from PDLLA: PEO nanofibers were recently published [7–9, 11–14].

Ciprofloxacin (CIP), a fluoroquinolone antibiotic, is commonly used in the treatment of *Pseudomonas* and *Staphylococcus* infections [4, 15], with few reports on the developing of resistant strains [4, 16, 17]. The electrospinning of CIP in dextran [18], poly(vinylalcohol) (PVA) [19], PVA/poly(vinyl acetate) [20], poly(L-lactide-co-D,L-lactide) [21], poly(4-vinylbenzoic acid-co-(ar-vinylbenzyl)trimethylammonium chloride) [22] and polydioxanone [23] were reported. No reports were found on the incorporation of CIP in PDLLA: PEO. In the present investigation, CIP was incorporated into a blend of PDLLA and PEO and electrospun into nanofibers (CIP-F). The effect of CIP-F on the formation of biofilms by *P*. *aeruginosa* PA01 and *S*. *aureus* Xen 30 is reported.

## Materials and Methods

### Bacterial strains and growth conditions

*Pseudomonas aeruginosa* PA01, containing the *gfp* gene, was grown in sterile tryptone soy broth (TSB, Biolab, Biolab Diagnostics, Midrand, South Africa). The methicillin-resistant *S*. *aureus* strain Xen 30, derived from the clinical strain *S*. *aureus* I6 (Caliper Life Sciences, USA), was cultured at 37oC in brain heart infusion (BHI) broth (Biolab).

### Determination of the minimum inhibitory concentration (MIC) of CIP

The MIC of CIP was determined using agar dilution plates, as described by Andrews [24]. CIP dilutions up to 128 mg l^–1^ were prepared. Overnight-grown cell suspensions of *P*. *aeruginosa* PA01 and *S*. *aureus* Xen 30 were diluted to represent 1 × 10^6^ CFU ml^-1^. Ten μl of each cell suspension was inoculated onto the surface of Mueller-Hinton (MH) agar (Sigma-Aldrich, USA), supplemented with a specific CIP concentration. The MIC of CIP was defined as the lowest concentration in the agar medium that prevented growth.

### Incorporation of CIP in PDLLA:PEO

CIP (15 mg), 120 mg PDLLA (Mw 75 kDa—120 kDa) and 120 mg (w/v) PEO (Mw 200 kDa) were suspended into 1 ml 2-chloroethanol. Nanofibers were spun from this solution according to Heunis et al. [13] and then dried under vacuum at 25°C for 48 h. Nanofibers without CIP served as control.

### Characterization of nanofibers

The nanofibers were gold-coated, as described by Heunis et al. [13] and the surface structure of the nanofibers studied using a scanning electron microscope (SEM, Leo 1430VP, Zeiss, Cambridge, England). From these images, the diameter of the nanofibers was calculated using ImageJ Software, version 1.46, Scion Corporation [14]. Protrusions from the nanofibers were visualised by transmission electron microscopy (TEM), using a Philips Tecnai TF20 (FEI, OR, USA). Surface topology was studied using a Nanosurf atomic force microscope (AFM) Easyscan 2 (Nanosurf Inc., CA, USA). Interactive properties of CIP, PDLLA and PEO were studied using a Fourier transform infrared (FTIR) spectroscope (Thermo Nicolet Avatar 330, Thermo Scientific, Waltham, MA, USA), equipped with a Smart Performer Zn/Se ATR (attenuated total reflection) accessory. Crystal formation and phase compositions were observed by X-ray diffraction (XRD), using a Bruker AXS D8 Advance X-ray diffractometer (Bruker AXS, Frankfurt, Germany) operated in locked coupled mode. The instrument was equipped with a Vantec-1 position sensitive detector optimized for Cu-Kα radiation at λ = 1.5406 Å. The X-ray tube was operated at 40 mA and 40 kV. Readings were recorded at a scanning rate of 1 sec/step, with a step size of 0.0275° in a 2θ range that extended from 4° to 69.99°.

### Release of CIP from nanofibers

Directly after electrospinning, 2.0 mg nanofiber disks, containing 66.0 μg CIP (CIP-F), were submersed in separate 1 ml sterile PBS (pH 7.3) and incubated at 37oC. One set of three disks were immediately transferred to 1 ml sterile PBS and the CIP in PBS measured by recording absorbance readings at 275 nm with a Cary 1E UV-VIS Spectrophotometer (Varian, USA). The other disks (three per set) were transferred to 1 ml sterile PBS after 2, 3, 4, 6, 24 and 48 h of incubation and the CIP released in PBS during incubation determined in the same way. Nanofiber disks without CIP (CF) served as control. Readings were compared to absorbance readings obtained for known CIP concentrations. Each experiment was performed in triplicate.

In a separate experiment, CIP-F disks of 2.0 mg were placed on the surface of two Mueller-Hinton (MH) agar plates. One plate was imbedded with an overnight grown culture containing 10^5^ CFU ml^–1^ *P*. *aeruginosa* PA01 and the other with an overnight grown culture containing 10^5^ CFU ml^–1^ *S*. *aureus* Xen 30. CF disks of the same weight served as control. All the plates were incubated at 37°C for 24 h. Disks surrounded with inhibition zones were aseptically removed from the plates, transferred to a new set of MH agar plates imbedded with cells and incubated for another 24 h. Disks were transferred every 24 h onto freshly seeded MH agar plates until no growth inhibition zones were observed. The diameter of each inhibition zone was expressed in mm.

In another experiment, 0.5 cm^2^ CIP-F disks were placed into 1 ml cell suspensions (log_10_ 7 CFU ml—^1^) of overnight-grown cultures of *P*. *aeruginosa* PA01 and *S*. *aureus* Xen 30, respectively. The cell suspensions were incubated for 24 h at 37oC. At specific time intervals, cell density readings were recorded at 600 nm. The experiment was repeated with 0.5 cm^2^ CF disks. Cell suspensions without nanofibers served as control.

### Exposure of biofilms to CIP-F and CF

CIP-F disks (0.5 cm^2^) were placed in the first 12 wells of a 48-well polystyrene flat-bottom multidish (BioLite MultiDishes, Thermo Scientific, NY). CF disks of the same size were placed in 12 other wells and another 12 wells received no disks. Overnight-grown cells of *P*. *aeruginosa* PA01 and *S*. *aureus* Xen 30 were diluted in sterile tryptone soy broth (TSB) to log _10_ 7 CFU ml^–1^. One ml cell suspension of *P*. *aeruginosa* PA01 was added to the first six of the 12 wells with no disks and to the first six of the two sets of 12 wells containing CIP-F and CF, respectively. The remaining wells each received 1 ml *S*. *aureus* Xen 30. All plates were statically incubated at 37oC for 48 h. At specific time points, planktonic cells were removed from the wells and the wells were carefully rinsed with sterile distilled water. The biofilms that formed in the wells were analysed for the presence of viable cells by plating onto Mueller-Hinton (MH) agar. The total number of cells, dead and viable, in the biofilm was recorded by staining with crystal violet [7, 8]. Absorbance of crystal violet was measured at 550 nm against 30% (v/v) acetic acid that served as blank. Microscopic images of the biofilms were recorded using a Carl Zeiss Binocular Microscope (Carl Zeiss, Germany), fitted with an external Olympus digital camera (15 MP, Olympus Corp., Japan).

### Determination of the number of viable cells in a biofilm

At specific time points, planktonic cells were removed from the wells, and all the wells were washed twice with sterile distilled water. Sterile 100 μl PBS (pH 7.3) was added to each of the wells and the cells in the biofilm suspended by using a sterile glass rod, followed by gentle vortexing for 2 min. The cell suspensions were serially diluted in PBS, plated onto tryptone soy agar (Biolab) and the number of colonies counted after 24 h of incubation at 37°C.

### Testing for exopolysaccharides and proteins in a biofilm

Exopolysaccharides (EPS) were isolated from 48 h-old biofilms, according to the method described by Zhao and Liu [25]. In brief, cold absolute ethanol was added to a biofilm to precipitate the EPS. The precipitate was dehydrated with 77.0% (v/v) sulfuric acid. Cold tryptophan (1.0%, w/w) was added and absorbance readings were taken at 490 nm. The concentration of EPS was determined by comparing the absorbance readings against readings obtained with known concentrations of pure dextran (mw 71.4 kDa, Sigma-Aldrich, USA). Protein concentrations in the biofilm were determined by using the Micro Bicinchoninic Acid (BCA) kit (Pierce, Rockford, IL).

### Cytotoxicity studies

Breast epithelial cells (MCF-12A) were cultured in 1 ml of a combination of Dulbecco's modified Eagle's medium (DMEM) and Hams-F12 (1:1), supplemented with 10% fetal bovine serum (FBS), 0.5 μg ml^–1^ hydrocortisone, 10 μg ml^–1^ insulin, 100 ng ml^–1^ cholera toxin and 1% PenStrep (all chemical from Sigma, USA). The cells were incubated at 37°C in humidified atmosphere and in the presence of 5% CO_2_. The MCF-12A cells were seeded in 12 × 24-well tissue culture plates (NEST, Nest Biotech, China) at 8.0 × 10^4^ cells per well. After 24 h the medium (1 ml) in each well was replaced with CIP-F (0.25 cm^2^), CF (0.25 cm^2^), 1.0% (v/v) Triton X-100 that served as positive control (PC) and fresh culture media that served as negative control (NC), respectively, and incubated for a further 24 h. MCF-12A cells were stained with MTT 3-(4,5-dimethylthiazol-2-yl)-2,5-diphenyltetrazolium bromide and absorbance readings were recorded at 595 nm. All experiments were performed in triplicate.

### Statistical analysis

GraphPad Prism [version 6.03 (Trial) for Windows, GraphPad Software Inc, USA] was used to perform one-way analysis of variance (ANOVA). *P* value < 0.05 was considered statistically significant.

## Results

### MIC of CIP

The MIC of CIP was 4 mg l^-1^ against 1 x 10^6^ CFU ml^-1^ *P*. *aeruginosa* PA01 and 16 mg l^-1^ against 1 x 10^6^ CFU ml^-1^ *S*. *aureus* Xen 30.

### Characterization of CIP-F

**Fig 1:**
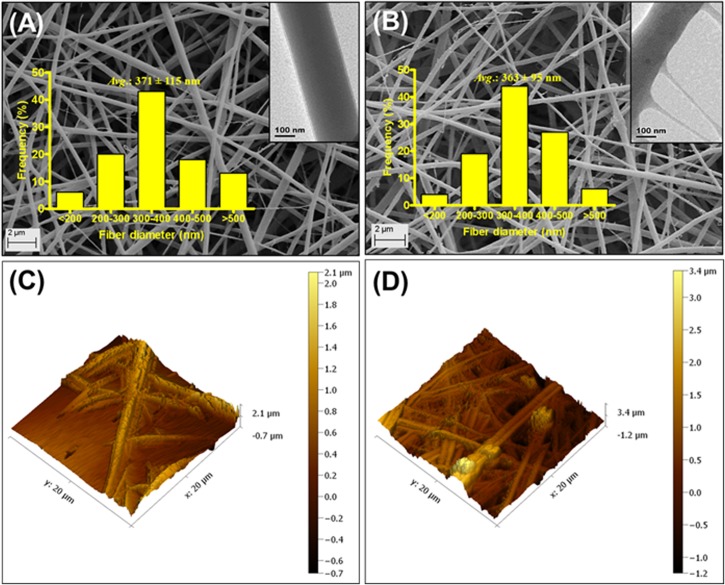
Scanning electron microscopy (SEM) images of A: nanofibers without ciprofloxacin, CIP (CF) and B: CIP-containing nanofibers (CIP-F). Images obtained with transmission electron microscopy (TEM) are shown as inserts. C and D: Atomic force microscopy (AFM) images of CF and CIP-F, respectively.

Nanofibers without CIP (CF) were slightly larger in diameter (371 ± 115 nm, Fig 1 A) than CIP-F (363 ± 95 nm in diameter, Fig 1B). Both nanofibers displayed a smooth surface when studied under the SEM (Fig 1A and 1B). However, images recorded with TEM showed a less uniform surface structure for CIP-F (Fig 1B, inserted image) compared to CF (Fig 1A, inserted image). The topography of CF and CIP-F, observed with AFM, were very similar and no crystal formation was visible (Fig 1C and 1D, respectively). CF had a root mean square roughness of 168 nm and a roughness average of 141 nm, whereas values recorded for CIP-F were 166 nm and 142 nm, respectively.

**Fig 2:**
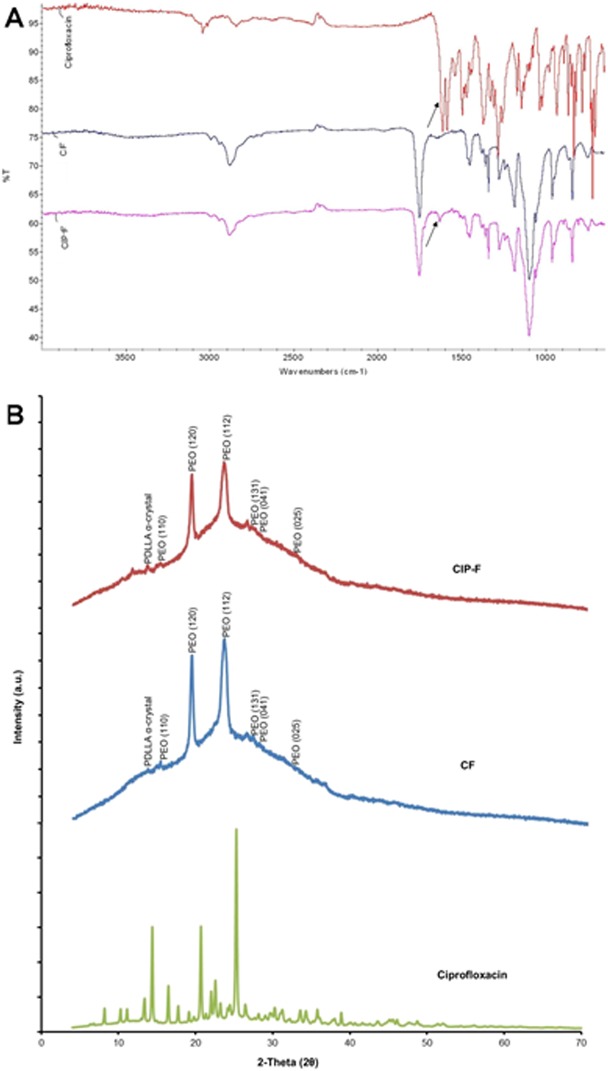
A: Fourier transform infrared (FTIR) spectra of ciprofloxacin (CIP), nanofibers without CIP (CF) and CIP-containing nanofibers (CIP-F). The arrows point to vibration peaks at 1632 cm^-1^, characteristic of C = O carbonyl bonds. B: X-ray diffraction (XRD) patterns of crystalline CIP, CF and CIP-F.

The FTIR vibration peaks recorded for CF and CIP-F at 2882 cm^-1^and 1752 cm^-1^ (Fig 2A) are characteristic of CH_3_ and (C = O)_ester_ bonds, respectively. Vibration peaks recorded at 1277 cm^−1^ and 1185 cm^−1^ (Fig 2A) are typical of C–O bonds. The scissoring and rocking modes recorded at 1453 cm^-1^ and 748 cm^−1^ (Fig 2A) are characteristic of CH_2_. All of these peaks are characteristic of PDLLA: PEO. An additional vibration peak was recorded at 1632 cm^-1^ for CIP-F (Fig 2A).

With XRD analysis, crystalline CIP showed characteristic peaks at 2θ values of 14, 20 and 25^o^ (Fig 2B). No diffraction peaks of crystalline CIP were detected in CIP-F (Fig 2B). The α crystal peak of PDLLA was observed at a 2θ value of 11^o^ (Fig 2B). Peaks at 14, 19, 23, 25, 26 and 32^o^ (Fig 2B) corresponded well with planes (110), (120), (112), (131), (041) and (025) of PEO.

### Release of CIP from CIP-F

**Fig 3:**
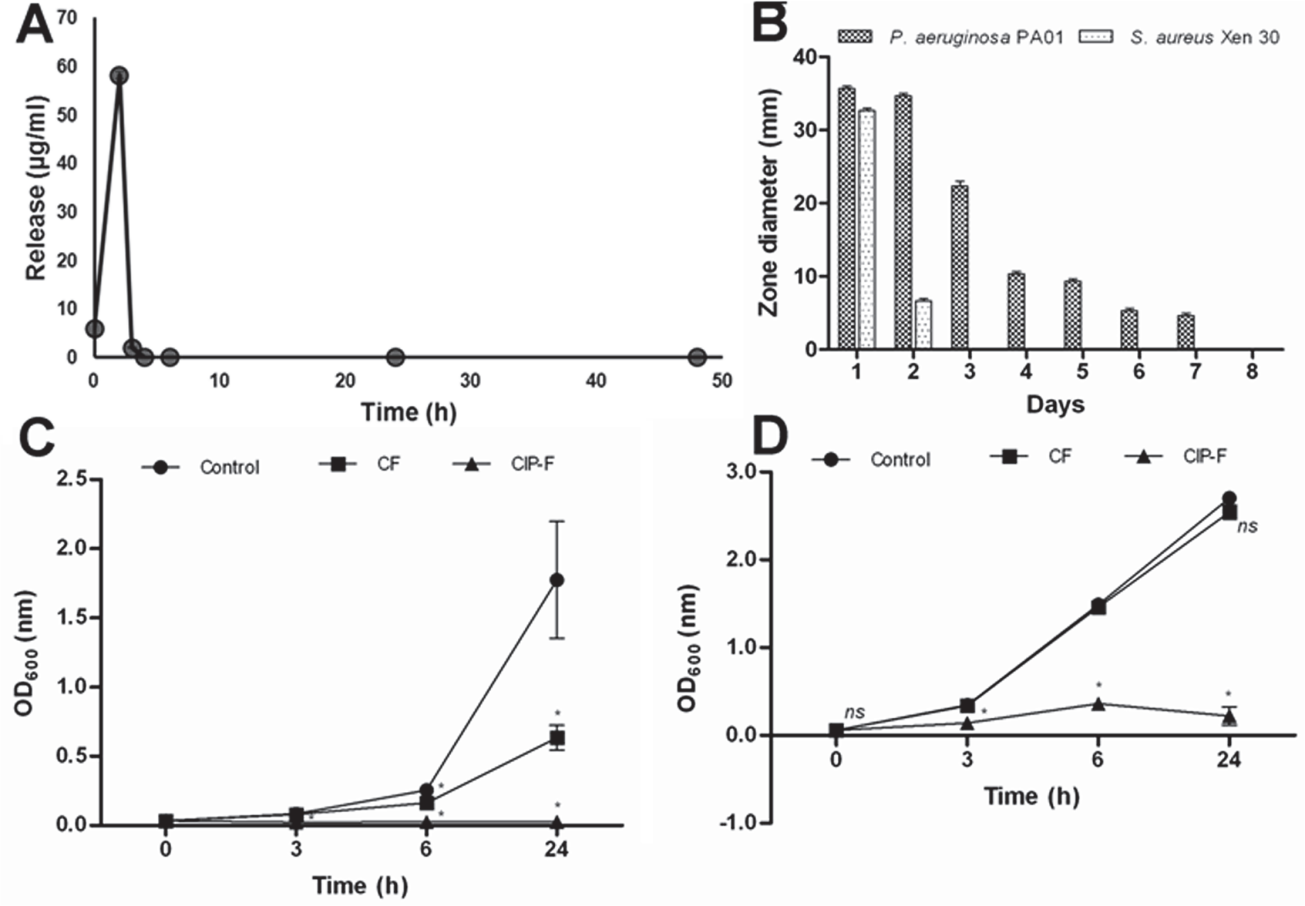
A: Release of ciprofloxacin (CIP) from PDLLA: PEO nanofibers immediately after immersion into PBS, and 2, 3, 4, 6, 24 and 48 h thereafter, B: *in vitro* antimicrobial activity of CIP-containing nanofibers (CIP-F) against *P*. *aeruginosa* PA01 and *S*. *aureus* Xen 30, C: cell density of *P*. *aeruginosa* PA01 exposed to nanofibers without CIP (CF) and D: cell density of *S*. *aureus* Xen 30 exposed to CF and CIP-F. The control was without nanofibers and without CIP. Data points represent an average reading recorded from three disks per time point and three independent experiments (mean ± standard deviation). * *p* < 0.05. *ns*: not significant.

Immediately after electrospinning, 6.01 μg CIP was released from 2 mg CIP-F (Fig 3A). Two hours later, 58.22 μg CIP was released (Fig 3A). The release of CIP decreased to 2.02 μg after a further 3 h (Fig 3A). All of the CIP was released within the first 3 h (Fig 3A). Despite the rapid diffusion of CIP from CIP-F, growth of *P*. *aeruginosa* PA01 was inhibited for seven consecutive days, as observed after seven transfers of CIP-F disks onto seeded plates (Fig 3B). Growth of *P*. *aeruginosa* PA01 was severely inhibited within the first 24 h, as shown by an inhibition zone of 35 mm in diameter (Fig 3B).

Growth of *S*. *aureus* Xen 30 was inhibited for 48 h only, with the largest inhibition zone (32 mm) recorded within the first 24 h (Fig 3B). Cell density of *P*. *aeruginosa* PA01 recorded when incubated in the presence of CF and in the absence of nanofibers (control) were more-or-less similar for the first 6 h. Optical density increased rapidly thereafter in the control (from OD_600_ 0.3 to 1.7) within the following 18 h, but increased less drastically in the presence of CF (from OD_600_ 0.2 to 0.6) over the same period (Fig 3C). Cell density was close to zero when cells were exposed to CIP-F and remained at this level for 24 h (Fig 3C). Growth of *S*. *aureus* Xen 30 were less affected by CF, as shown by a drastic increase in cell density; from OD_600_ 0.2 after 3 h to approximately 2.5 after 24 h (Fig 3D). The cell density of *S*. *aureus* Xen 30 remained below OD_600_ 0.2 when cells were exposed to CIP-F (Fig 3D). No change in the appearance of the nanofibers was observed with the release of CIP or when the nanofibers came into contact with bacterial cells. This is in agreement with a previous study that showed a large degree of miscibility between PEO and PDLLA and stability in structure when the two polymers were used in a 50:50 combination [12].

### Effect of nanofibers on biofilm formation and cell viability

**Fig 4:**
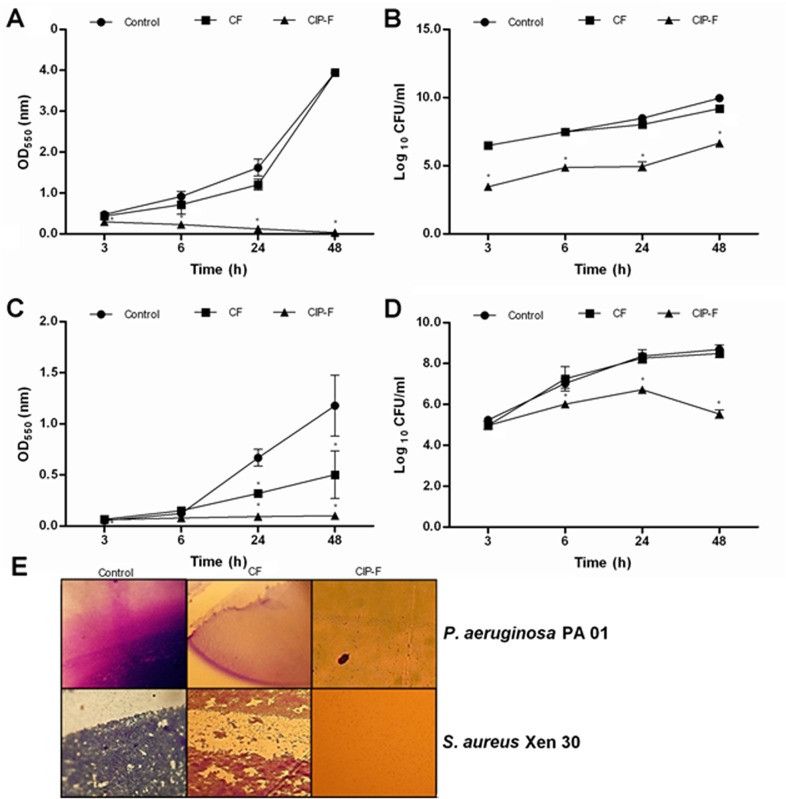
A and C: Biofilm formation recorded for *P*. *aeruginosa* PA01 and C: *S*. *aureus* Xen 30, respectively. Biofilm formation is expressed as optical density of crystal violet-stained cells. B and D: viable cell numbers recorded for *P*. *aeruginosa* PA01 and C: *S*. *aureus* Xen 30, respectively. E: Biofilm images recorded with a light microscope. CIP-F = nanofibers containing ciprofloxacin (CIP), CF = nanofibers without CIP, control = no nanofibers and no CIP. Data points presented are the average of three independent experiments (mean ± standard deviation). * *p* < 0.05.

*P*. *aeruginosa* PA01 formed a strong biofilm over 48 h in the presence of CF, as indicated by an increase in OD_550_-readings from 0.5 to 4.0 (Fig 4A). Changes in OD-readings were very similar to that recorded for biofilm formation in the absence of nanofibers, i.e. the control (Fig 4A). A steady increase in cell numbers (from log _10_ 7 CFU ml^–1^ to approximately log _10_ 9 CFU ml^–1^) was recorded in both these biofilms (Fig 4B). Biofilm formation was inhibited (and even declined) when cells were exposed to CIP-F (Fig 4A). However, an increase in viable cell numbers was recorded (from log _10_ 3 CFU ml^–1^ to log _10_ 6 CFU ml^–1^) in biofilms exposed to CIP-F (Fig 4B). Microscopic images taken of the *P*. *aeruginosa* PA01 biofilms after 48 h of incubation is shown in Fig 4E.

In comparison to *P*. *aeruginosa* PA01, *S*. *aureus* Xen 30 formed a much weaker biofilm, as shown by an increase in OD_550_-readings from 0.1 to 1.2 over 48 h (Fig 4C). Exposure to CF over the same period weakened biofilm formation, with an OD_550_-reading of 0.5 recorded after 48 h (Fig 4C). However, the number of viable cells in CF-exposed and in the control biofilms increased from log _10_ 5 CFU ml^–1^ to log _10_ 8 CFU ml^–1^ over 48 h (Fig 4D). Cell numbers in biofilms exposed to CIP-F increased from log _10_ 5 CFU ml^–1^ to log _10_ 6 CFU ml^–1^ for the first 24 h, but decreased to log _10_ 5 CFU ml^–1^ over the next 24 h (Fig 4D). Microscopic images taken of the *S*. *aureus* Xen 30 biofilms after 48 h of incubation is shown in Fig 4E.

### EPS and protein content in biofilms

The EPS and protein concentrations in *P*. *aeruginosa* PA01 biofilms exposed to CF were 112 μg ml^–1^ and 7.6 μg ml^–1^, respectively. Higher EPS (165 μg ml^–1^), but lower protein (5.8 μg ml^–1^) concentrations were recorded for biofilm not exposed to nanofibers (control). No EPS, nor proteins, were detected in biofilms exposed to CIP-F. Similar results were recorded for *S*. *aureus* Xen 30 biofilms. Protein concentrations decreased from 10.3 μg ml^–1^ for the control to 4.9 μg ml^–1^ when exposed to CF, and to no detectible levels when exposed to CIP-F. No EPS production was detected in any of the *S*. *aureus* Xen 30 biofilms.

### Cytotoxicity evaluation

**Fig 5:**
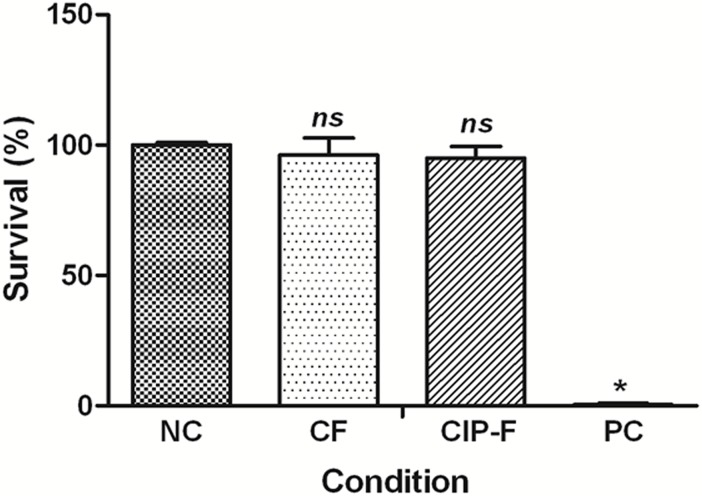
Survival of MCF-12A breast epithelial cells after treatment with ciprofloxacin (CIP)-containing nanofibers (CIP-F) and nanofibers without CIP (CF). Freshly prepared culture medium served as a negative control (NC) and Triton X-100 as positive control (PC). Data points presented are the average of three independent experiments (mean ± standard deviation). * *p* < 0.05. *ns*: not significant.

No significant difference in cell viability was observed when breast epithelial cells MCF-12A were exposed to CF, CIP-F and in the absence of nanofibers (Fig 5). Almost no cells survived exposure to Triton X-100 (Fig 5).

## Discussion

The small variations in diameter recorded for nanofibers without CIP (CF) and those containing CIP (CIP-F), shown in Fig 1A and 1B, may be ascribed to small changes in viscosity and conductivity of the PDLLA: PEO solution. These results are in agreement with our previous findings when 2,3-dihydrobenzoic acid and nisin were electrospun into PDLLA: PEO [7, 8, 11–14]. This may be characteristic for PDLLA: PEO nanofibers only. Incorporation of CIP into poly(4-vinylbenzoic acid-co-(ar-vinylbenzyl)trimethylammonium chloride) increased the diameter of nanofibers [22]. The smooth surfaces, absence of bead formation (SEM images, Fig 1A and 1B) and lack of crystal formation (AFM, 1C and 1D) suggests that CIP is completely miscible with PDLLA and PEO. This was confirmed by similar values recorded for root mean square roughness (166 for CIP-F and 168 for CF) and roughness average (142 for CIP-F and 141 for CF) obtained by AFM and similar FTIR profiles recorded for CF and CIP-F (Fig 2A). The only difference in FTIR profiles of CF and CIP-F was the vibration peak recorded at 1632 cm^-1^ (Fig 2A), which is typical of the C = O _carbonyl_ bond present in CIP [22]. Detection of CIP as a single peak suggests that the antibiotic did not react with PDLLA or PEO and that it could be released in an active form [22]. No diffraction peaks were recorded for CIP in CIP-F (Fig 2B), suggesting that the antibiotic is entrapped in the polymer in its crystalline form [22], without forming interactions with PDLLA and PEO. The protrusions observed on CIP-F (Fig 1B insert image) may be CIP adhered to the surface of the nanofibers. Overall findings indicated that CIP remained intact in the nanofiber matrix and did not form an irreversible reaction with PDLLA and PEO, suggesting that it could be released from the nanofibers in an active form. No significant structural changes or signs of deterioration were observed when nanofibers prepared with a 50:50 blend of PEO and PDLLA were exposed to PBS buffer (pH 7.4) for 8 days [12], suggesting that the polymers did not leach or degrade during these experiments.

Most of the CIP was released from CIP-F within 2 h (64 μg of the 66 μg; Fig 3A), killing all cells of *P*. *aeruginosa* PA01 in a liquid culture (Fig 3C) and most cells imbedded in an agar medium (Fig 3B). The initial burst release of CIP from PDLLA: PEO nanofibers is similar to the release of nisin from the same nanofibers [13]. Prolonged inhibition of *P*. *aeruginosa* PA01 after all CIP was released from the nanofibers suggests that the initial burst release of the CIP repressed growth of the few remaining viable cells for at least a further 22 h.

Exposure of cells to CIP-F on solid growth medium mimicked conditions encountered in topical infections. Based on the agar diffusion test, most of the CIP diffused from the nanofibers within the first 2 days, but concentrations remained high enough (above the determined 4.0 mg l^-1^) to control cell growth for 5 days longer (Fig 3B). Although biofilm formation was prevented for 48 h when cells were exposed to CIP-F (Fig 4A), the number of viable cells increased from log _10_ 3 CFU ml^–1^ to log _10_ 6 CFU ml^–1^ (Fig 4B) within the same period. This suggested that the viable cells lacked the ability to form a biofilm.

Almost all cells of *S*. *aureus* Xen 30 were killed within 2 h of exposure to CIP that diffused from CIP-F (Fig 3B and 3D). However, after 2 days of exposure to CIP-F, no growth inhibition zones were observed (Fig 3B). This suggested that *S*. *aureus* Xen 30 became resistant to CIP or that the levels were below the determined MIC of 16.0 mg l^-1^. Biofilm formation was prevented for 48 h when cells were exposed to CIP-F (Fig 4C), but cell numbers increased from log _10_ 5 CFU ml^–1^ to log _10_ 6 CFU ml^–1^ for the first 24 h. The following decrease in *S*. *aureus* Xen 30 cell numbers (Fig 4D) is either an indication that the few remaining cells in the biofilm lost the ability to support quorum sensing, or that quorum sensing (that may be involved in biofilm formation) is only supported when cell numbers are below log _10_ 6 CFU ml^–1^.

The rapid release of CIP from CIP-F is similar to release studies reported for most quinolone antibiotics [26]. The rate at which a drug is released from its matrix is important in control of infection [13]. The high surface-to-volume ratio of nanofiber wound dressings offers rapid hydration of hydrophilic material. The shorter diffusion distance creates a concentration gradient of encapsulated molecules, which further enhances the rate of mass transport into mucosal tissues [9, 27]. Diffusion of CIP into a biofilm depends on the net charge of the biofilm and that of CIP. At a neutral pH, CIP exists primarily as a zwitterion [28]. However, at lower pH, CIP is positively charged and entering into bacterial cells is restricted [28]. Thus, under optimal pH conditions, such as physiological pH, penetration of CIP into a biofilm may be enhanced [16] and cells causing the infection may be eradicated more effectively. All studies conducted were in PBS at pH 7.3, which means CIP existed as a zwitterion.

Exopolysaccharide (EPS) and proteinaceous molecules of microorganisms play an important role in biofilm formation [29, 30]. In this study, no EPS and proteins were detected in biofilms exposed to CIP-F. This could explain why biofilms were not formed. Biofilm formation in infection sites is usually enhanced in the presence of EPS and proteins [25].

The fact that no significant differences were recorded in the viability of MCF-12A breast cells exposed to CF, CIP-F and in the absence of nanofibers (Fig 5) indicated that PDLLA: PEO may be a safe option to deliver CIP to sites of infection. The FDA already approved this safety of PDLLA and PEO in biomedical implants [9, 10].

## Conclusion

Ciprofloxacin was successfully encapsulated into PDLLA: PEO nanofibers. The amorphous distribution of CIP in nanofibers without noticeable chemical interactions, its ability to prevent biofilm formation by *P*. *aeruginosa* and *S*. *aureus* and safety against mammalian cells suggests that dressings produced from CIP-impregnated nanofibers may be used to prevent biofilm formation in topical infections caused by these bacteria.
